# Chromatographic Characterization and Method Development for Determination of Levetiracetam in Saliva: Application to Correlation with Plasma Levels

**DOI:** 10.1155/2017/7846742

**Published:** 2017-08-07

**Authors:** Imad I. Hamdan, Mervat Alsous, Amira Taher Masri

**Affiliations:** ^1^School of Pharmacy, The University of Jordan, Amman, Jordan; ^2^Department of Clinical Pharmacy and Therapeutics, Faculty of Pharmacy, Applied Sciences Private University, Amman, Jordan; ^3^Faculty of Medicine, The University of Jordan, Amman, Jordan

## Abstract

Levetiracetam (LVT) is a widely used antiepileptic drug (AED). A less invasive sampling method for therapeutic drug monitoring (TDM) would be very useful particularly for children. Saliva has been shown as an adequate sample for TDM of some AEDs. Due to the high hydrophilicity of LVT its separation on common stationary phases is quite a challenge so that previous methods for determination of LVT in saliva employed either gradient high performance liquid chromatographic (HPLC) system or mass spectrometer as a detector. In this study the retention behavior of LVT on some common stationary phases was examined, with C8 being the most retentive. A simple isocratic HPLC method that is based on simple protein precipitation was developed and validated for the determination of LVT in saliva. The method was applied to a sample group of epileptic children for the purpose of assessing potential correlation with plasma LVT levels and to investigate patient's compliance. The results confirmed a reasonable correlation between plasma and salivary levels of LVT (*R* = 0.9) which supports the use of saliva for TDM of LVT. The study also revealed a significant percentage of epileptic patients having LVT levels below the estimated therapeutic range.

## 1. Introduction

**Figure 1:**
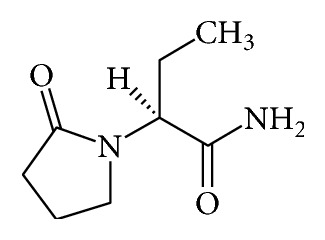
Structure of levetiracetam (LVT).

Levetiracetam (LVT) was approved by American Food and Drug Administration (FDA) as a second generation broad spectrum antiepileptic drug in 1999 [1]. LVT is structurally different from older antiepileptic generations with substantial hydrophilic substituents and no ionizable groups (Figure 1). Therefore, a pH independent high water solubility of 0.104 g/ml has been reported for the drug [2]. Therapeutic effectiveness of the drug in cases where older drugs fail has been established in a number of studies [3]. LVT is rapidly absorbed from gastrointestinal tract when given orally and excreted in the urine to a large extent with negligible affinity towards plasma protein [4]. Although LVT is generally a safe drug that might be given in oral doses of up to 3000 mg/day, some serious side effects might not be excluded at high daily doses [5]. Hepatic-enzyme-inducer drugs have been shown to increase the clearance of LVT to some extent, that is, shortening the half life, and in clinical use may lead to suboptimal therapeutic effect of LVT [6]. Therefore, due to high individual variability, it is usually recommended to perform therapeutic drug monitoring (TDM).

TDM is almost a routine for all other antiepileptic drugs. For LVT, quite a good number of studies have been published for determination of the drug in plasma. These include gas chromatography [7], capillary electrophoresis [8], ultraperformance liquid chromatography [9, 10], and liquid chromatography-mass spectrometry [11–13] but most of the published methods were based on HPLC-UV/or photodiode array detector (PDA) as the most widely available technique [14–20]. However, there were only three methods reported for the determination of the drug in saliva; the first of them was based on HPLC-UV with gradient elution [21] and the others were based on either gas chromatography tandem mass spectrometry (GC–MS) [22] or Liquid Chromatography tandem mass spectrometry (LC-MS) [12]. Using saliva drug level, as a quick simple and noninvasive method for TDM, offers substantial advantages for children and patients with poor venous access. Therefore it is important to have a reliable and simple analytical method for determination of LVT in saliva if a correlation was established between plasma and saliva concentrations. The only two reports that studied potential correlation of LVT concentration between plasma and saliva concluded good correlation coefficients in the range 0.86–0.9 and recommended potential use of saliva for TDM [21, 22].

The main challenge in the determination of LVT in biological fluids lies in its hydrophilic behavior, which leads to low retention on commonly employed reversed phase packing material [17, 18]. In some of the reported methods, this problem has been overcome using a gradient elution system [17, 20, 21]. In other reports, mass spectrometry was used as a detector with HPLC [11, 13] so there would be no worries about extent of drug retention, because of the high selectivity of the MS as a detector. If a simple isocratic system is sought, then a rather more polar stationary phase might have to be used so that it would provide reasonable retention for LVT. The reported data for retentivity of LVT were somehow conflicting. For example, two studies have utilized C18 columns with the same dimensions (250 mm), almost same flow rate and temperature, but utilized different mobile phases, namely, 30% acetonitrile (ACN) at pH 6.5 [23] and 10% ACN at pH 6.6 [9], and the reported retention times (*t*_*r*_) for LVT were ~10 and 6 min, respectively. Retention factors (*K*) for LVT could be roughly calculated from the provided chromatograms for the two conditions and found as 18 and 1.6, respectively. Thus instead of having higher *K* values for lower percentage of organic component, the estimated value was much lower.

Good number of the reported methods which employed HPLC systems recommended a rather high temperature (40–70°C) [10, 16, 23] which accords with the anticipated high diffusibility of LVT as a small molecule together with its interaction with the residual polar silanol groups. Additionally, some reports have utilized amine modifiers in the mobile phase such as (TEA) or dibutylamine [16, 18]. Amine modifiers are generally added to reduce peak broadening of basic analytes which usually result from their interaction with residual silanol groups [24].

Despite the high polarity of LVT, some reported methods utilized liquid-liquid extraction (LLE) with organic solvents such as ethyl acetate, chloroform, and dichloromethane for its extraction from plasma which might not provide the maximum recovery [10, 15, 16, 21]. Some authors have used NaOH [21] and others used HCl [16] to modify the pH of plasma during extraction in spite of the drug being, practically, neither an acid nor a base (pKa 16.1 and −1; ChemAxon). Interestingly, one report has shown that extraction of LVT from biological samples with cyclohexane would leave almost all of LVT in the aqueous phase while getting rid of the lipophilic constituents through the organic phase [17], in an obvious contradiction to the previous references. Even when solid-phase extraction (SPE) was employed, the samples were washed while on cartridges with water and eluted with methanol or even chloroform/isopropanol, which might not also be ideal because the drug is highly water soluble [20, 22]. Thus the reported recovery values for some of the SPE methods were below optimum [13, 20]. Indeed, one report has compared different methods (SPE and protein precipitation (PP)) for extraction of the drug from plasma samples and concluded that PP was significantly faster than SPE while providing comparable recovery. However, one potential problem might be associated with the former was the resultant dilution of the sample and the consequent loss in sensitivity [18].

Therefore, there is a need to properly characterize the chromatographic behavior of LVT on different chemistries of stationary phases in search for the optimum performance which might allow sufficient retention of the hydrophilic LVT while minimizing that of lipophilic analytes. In addition a validated simple isocratic HPLC-UV method that is systematically optimized based on theoretical understanding of LVT properties and in light of literature data is required.

In this study, we characterized the performance of LVT on a range of commonly available HPLC columns of different stationary phase chemistries and optimized systematically a simple method, based on PP, for determination of LVT in saliva. The method was applied to monitor LVT levels in saliva of a group of children who were clinically prescribed the drug. Moreover, attempts were made to assess potential correlation between LVT determined in saliva by the proposed method and that determined in plasma samples of the same patients using a validated HPLC method in accredited hospital laboratory [18].

## 2. Experimental

### 2.1. Reagents and Materials

LVT was purchased from Sigma Aldrich Company. HPLC grade ACN and methanol were from TEDIA (Tedia INC, USA). All other reagents were from Sigma (USA). Chromatographic system consisted of a Merck Hitachi HPLC unit equipped with a UV detector (set at 205 nm), water bath to control temperature, and a computer with Claritylight™ for data handling. The columns examined included standard C18 (250 mm × 4.6 i.d; 5 *μ*m; Hypersil®, Thermo-scientific), C8 (250 mm × 4.6 i.d; 5 *μ*m; Luna Phenomenex®), cyano (150 mm × 4.6 i.d; 5 *μ*m; Zorbax CN®; Agilent), phenyl-hexyl (250 mm × 4.6 i.d; 5 *μ*m; Hypersil™, Thermo-scientific), Synergi Polar RP (150 mm × 4.6 i.d; 4 *μ*m; Phenomenex), Diol (250 mm × 4.6 i.d; 5 *μ*m; LiChrospher® 100), and C1-SAS (250 mm × 4.6 i.d; 5 *μ*m; Hypersil, Thermo-scientific).

### 2.2. Chromatographic Conditions

As the study was concerned about chromatographic characterization of LVT, several stationary phases (columns) were examined using a series of mobile phases containing decreasing concentrations of ACN (80–3.5%) in phosphate buffer. The pH of the mobile phase was examined in the range 3.5–6.5. The finally recommended condition for the analysis of LVT in saliva was a C8 column with 3.5% ACN in 50 mM phosphate buffer at pH 6.0 as a mobile phase was pumped at a flow rate of 1.5 ml/min with the temperature of the column maintained at 41°C.

### 2.3. Preparation of Stock Solutions and Calibration Curves

Stock solutions of LVT and the internal standard (IS) 5,5-diethylbarbituric acid (DEBA) were prepared at concentrations of 0.48 and 0.5 mg/ml, respectively, in 80% ACN. From the prepared stock solution of LVT, different volumes were transferred to separate 10 ml volumetric flasks containing 1 ml of IS solution and volumes completed using 80% ACN. Thus 7 calibration concentrations were prepared to contain 50 *μ*g/ml of IS in addition to increasing concentrations of LVT in the range of 0.48–48 *μ*g/ml. From each of the calibration concentrations so prepared, 150 *μ*L was mixed with 150 *μ*L of blank saliva in Eppendorf tubes to mimic the preparation of the saliva samples to be analysed. Samples were vortex mixed for 1 min and centrifuged for 10 min; then 100 *μ*L of the supernatant was mixed with 100 *μ*L of the mobile phase and vortex mixed and 100 *μ*L of the resulting mixture was manually injected into HPLC system.

### 2.4. Analysis of LVT in Saliva Samples

For saliva, analysis proceeded as follows: 100 *μ*L of thawed saliva was vortex mixed for 1 min. 100 *μ*L of IS solution (100 *μ*g/ml in 40% ACN) was added to affect PP by ACN and the mixture was vortex mixed for 3 min and centrifuged for 10 minutes. 150 *μ*L of the supernatant was further diluted with 150 *μ*L of mobile phase, the mixture was vortex mixed, and 100 *μ*L of the resulting mixture was manually injected into HPLC system.

### 2.5. Validation Studies

Selectivity was assessed by obtaining chromatograms for blank saliva samples obtained from six different volunteers and processed using the same optimum conditions. Recovery was examined at low, mid, and high concentrations (0.24, 18, and 48 *μ*g/ml) by subjecting blank saliva samples spiked with the proper concentrations of LVT to the same sample preparation procedure. Precision (expressed as relative standard deviation (RSD)) and accuracy (expressed as percentage error) were determined for samples at low, mid, and high concentrations by calculating the concentrations of quality control samples (QC) using the obtained calibration curves. The limit of quantification (LOQ) was determined as the concentration in saliva (standard preparation in saliva) that produced a peak response that was 10 times the noise level.

### 2.6. Analysis of LVT in Plasma Samples

Blood samples (2 ml) were collected from each participating child at a clinic visit. All samples were labeled with patient study number and the date and time of collection. Plasma was obtained for each sample after blood centrifugation at speed of 4000 rotations per minute (rpm) for 10 min. Aliquots were frozen at −80°C until analysis was performed using a validated HPLC method [18]. Waters Oasis® HLB cartridges (30 mg, 1 ml) were used for sample clean-up. After conditioning with 2 ml methanol and equilibration with 2 ml water, 500 *μ*L plasma samples was loaded and then washed twice with 1 ml water and eluted with 1 ml methanol. The eluate was then dried under vacuum (rotary evaporator), then reconstituted in 500 *μ*L of the mobile phase and injected into Agilent HPLC equipped with a diode array detector set at 205 nm [18].

A ResElut reversed phase column (C8, 150 × 4.6 mm i.d., 5 *μ*m) was used. The mobile phase was (6 : 5 : 89, v/v/v) a mixture composed of methanol, ACN, and a 3 mM phosphate buffer, respectively, containing 0.5 ml of triethylamine, pH of 6.0. Flow rate was 1 ml/min and adenosine was used as IS.

### 2.7. Collection of Saliva and Plasma Samples

The study was approved by the Research Ethics Committees in University of Jordan Hospital (Reference number: 09/NIR03/74). A sample of 15 children was recruited at the pediatric epilepsy outpatient clinic at the University of Jordan Hospital. Patient recruitment was commenced from October 2015 to November 2016. The parents of all children (≤18 years) with a diagnosis of epilepsy who attended the participating clinic and who had been prescribed LVT for at least one month were invited to take part in the research. Children were included in the study only after their parents signed the study consent form after full explanation of the study. Once child and his/her parent were recruited, patients' demographic data, medical and medication history, current medications, lab tests, and seizure control were obtained from the patients' medical notes.

## 3. Results and Discussion

### 3.1. Optimization of Sample Solvent and Extraction Conditions

In preliminary phase of method development and using typical C18 HPLC columns, it was evident that LVT had no sufficient retention to allow proper separation from the rather complex sample matrix of saliva. Using C8 column, addition of trimethylamine (TEA) in concentrations of 0.5–1.5% did not seem to bring about any improvement regarding the peak shape of LVT, which was not really surprising as LVT did not have a substantial basic properties. The initially observed peak of LVT, when dissolved in pure ACN, suffered extreme fronting which was most likely a result of sample solvent being much stronger eluent than the mobile phase (10% ACN in phosphate buffer, pH 6, column C8). When LVT was dissolved in the same mobile phase, the peak shape improved significantly. Peak shape remained reasonable up to 30% ACN as a sample solvent; that was taken into consideration when optimizing sample preparation.

Regarding sample preparation, PP has been previously employed in the extraction of LVT from plasma with satisfactory results [11, 12, 14, 23]. Therefore, PP was adopted as the extraction technique in this study so that the method would be kept simple, nonexpensive, and recovery maximized, in addition to being more consistent with the theoretical principles pertaining physicochemical properties of LVT. In order to overcome the potential sample dilution effect, a larger sample volume was injected, that is, 100 *μ*L, which is 10–20 times higher than those employed in previous studies. ACN was chosen for PP but our preliminary experiments showed that high percentages of ACN in the sample resulted in a significant peak fronting. Therefore, sample preparation was decided to be carried out by adding equal volumes of 80% ACN to saliva (100 *μ*L) and after centrifugation the supernatant was further diluted with the mobile phase (3.5% ACN) in 1 : 1 ratio so that the final percentage of ACN in the sample was <25%.

### 3.2. Chromatographic Characterization and Column Selection

**Figure 2:**
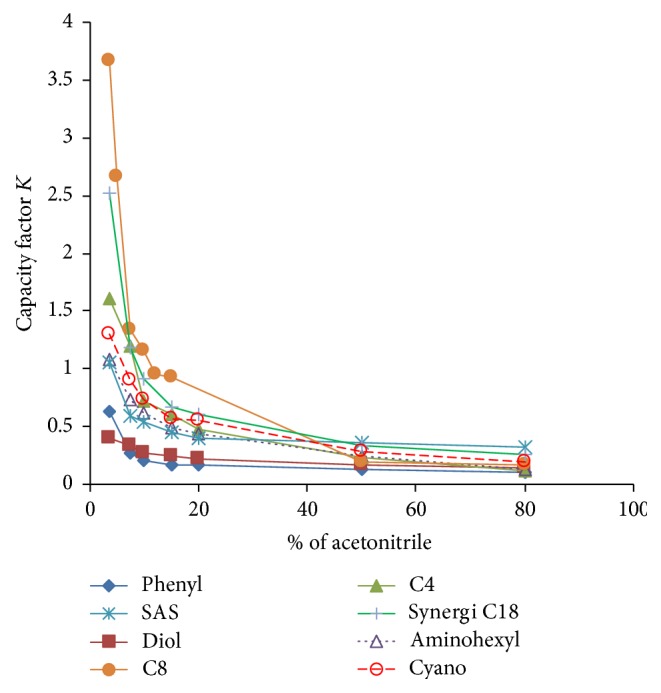
Plot of capacity factor “*K*” obtained for LVT using different columns against percentage of ACN in the mobile phase.

**Figure 3:**
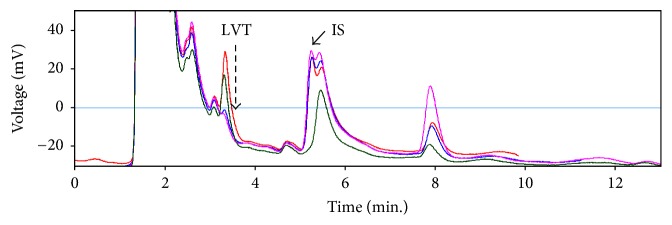
Chromatograms for saliva samples spiked with DEBA (internal standard, IS) and decreasing levels of LVT according to the direction of the dashed arrow (36, 28, 4, and zero *µ*g/ml). Note the overlapping with the peak of IS and at about 5.8 min. In the sample spiked with 28 *µ*g/ml of LVT no DEBA was added. Column: Synergi Polar RP (150 mm × 4.6 i.d). Mobile phase was 3.5% ACN in phosphate buffer at pH 6 with a flow rate of 1.5 ml/min.

Using a standard solution of LVT at 48 *μ*g/ml in 22% ACN the *t*_*r*_ values for LVT on different columns were determined in mobile phases that contained different percentages of ACN at pH 6.0. Using the solvent front peak as a measure of *t*_*r*_ for unretained analytes (*t*_*o*_), the capacity factors (*K*) were determined in each case. A plot of *K* against the percentage of organic solvent for the various columns is shown in Figure 2. Because the employed columns were of different dimensions, *K* values rather than *t*_*r*_ were employed to compare retentivity of the analyte because they are known to be less dependent on column dimensions and other physical parameters. All tested columns exhibited increase in their *K* values at lower percentages of ACN; particularly at percentages < 10%. It was rather surprising that the hydrophilic diol columns exhibited the least retentivity. Considering the recommended optimum *K* values as 0.5–20 [25], only three stationary phases provided reasonable values of *K*. The most retentive stationary phases at low percentages of ACN were C8, Synergi Polar RP, C4, and to some extent cyano, with C8 exhibiting remarkably higher retentivity. The performance of the three most retentive columns (C8, Synergi Polar RP, and C4) for the determination of LVT in saliva was evaluated using the lowest tested percentage of ACN (3.5%) and DEBA as an IS. For Synergi and C4 columns, the obtained *t*_*r*_ values for LVT were low (3.4–3.7), and there appeared to be serious overlapping with some saliva components particularly at low concentrations as shown for Synergi column (Figure 3). Yet more serious overlapping was observed between some saliva components and the employed IS at about 5.8 min (Figure 3).

Therefore C8 was decided to be the optimum stationary phase material to be employed for further method development. Highest *t*_*r*_ values (~9.8 min) for LVT were obtained on C8 column when ACN at a percentage of 3.5% was employed and that percentage was decided to be adopted for further method development because higher retention would result in higher probabilities of separation. It is noteworthy that the employed C8 column was of longer dimensions than some of other tested columns which contributed to the obviously longer *t*_*r*_ values. However, dimensions of columns are not direct determinants of *K* which is more suitable measure of retentivity of the analyte. In this study, some longer columns (SAS) provided only slightly longer *t*_*r*_ of LVT, but obviously lower *K* values than some other shorter columns (Synergi).

A guard column manually filled with C18 packing material was used to retain the more lipophilic natural constituents of saliva. As lipophilic constituents would not be eluted using the low elutropic 3.5% ACN mobile phase, they were accumulated on the C18 guard column and thus did not interfere with the subsequent injections. However, the guard column was refilled every about 50 injections.

Few compounds were tested for their potential use as IS including gabapentin and the amino acid methionine and DEBA. Since DEBA eluted at a reasonably longer *t*_*r*_ than LVT, allowing higher potential of resolution from copresent substances, it was adopted as IS.

### 3.3. Optimization of the Chromatographic Separation

**Figure 4:**
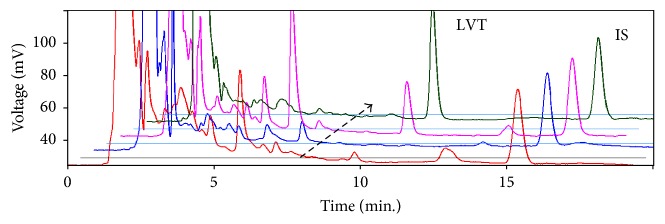
Selected chromatograms for samples of saliva spiked with IS and LVT as follows (according to the direction of the arrow): (1) blank saliva spiked with LVT at LOQ (0.24 *µ*g/ml); (2) blank saliva showing no interference with LVT; (3) and (4) are actual saliva of patients showing different levels of LVT, that is, 11.4 and 24.6 *µ*g/ml. The column was C8 using the finally recommended conditions.

Chromatograms were obtained for saliva samples spiked with LVT and DEBA as well as blank saliva samples (from 6 different volunteers) to check for any potential overlapping or interferences. Very good separation of LVT (at ~9.8 min) and IS (at 15.1 min) from the sample components was achieved (Figure 4). Despite the rather long run time due to the IS, no attempts were made to decrease run time further as that could jeopardize the separation and quantification of LVT particularly at low concentrations. As no indications of serious interferences were observed; the method was concluded selective and that condition was adopted as the optimum: 3.5% ACN in 50 mM phosphate buffer at pH 6, temperature 41°C, and a flow rate of 1.5 ml/min.

Interestingly, several other peaks could be observed separated in the chromatogram alongside LVT. That was important since one of our objectives was to attempt to improve correlation between levels in plasma and saliva possibly through normalizing the found concentration (or peak area) of LVT against peak area for some physiological compounds or LVT metabolites. A potential peak that appeared promising for that purpose was that at ~5.9 min (Figure 4) which appeared in several chromatograms of patients and somewhat was inversely proportional to the peak area of LVT. However, several attempts were made to normalize the concentration of LVT against that peak, for example, dividing the area of LVT by that at 5.9, but results were not satisfactory.

### 3.4. Validation: Linearity, Precision, and Accuracy

Linearity was established in the examined range of 0.24–48 *μ*g/ml with good correlation coefficients obtained (>0.992). A typical calibration equation was given by *A* = 0.07*x* + 0.024 where *A* is the peak area ratio of LVT/DEBA, and *x* is the concentration of LVT in *μ*g/ml. LOQ was accepted as 0.24 *μ*g/ml because that was the lowest concentration examined and provided a signal to noise ratio of higher than 10 and RSD values <8%. The achieved LOQ represents a reasonable improvement over some of previously reported methods where LOQ values ranged between 0.5 and 2 *μ*g/ml [17]. Surely the large injection volume employed in this study (100 *μ*L) has contributed to the achieved LOQ.

Recovery, accuracy, and precision were determined for quality control (QC) samples of low, mid, and high concentrations (0.24, 18, and 48 *μ*g/ml); a summary of the so obtained results is presented in Table 1. According to data presented in Table 1, it was evident that the method demonstrated satisfactory precision accuracy and recovery. The obtained values for recoveries were higher than those previously published using other extraction techniques and accord better with the fact that LVT is a hydrophilic nonionizable and non-protein bound compound. A more detailed comparison of the performance of the proposed method with those reported for determination of LVT in saliva is presented in Table 2. Generally the proposed method could favorably compare with the reported ones. Thus a simple isocratic method that needs no SPE or LLE but a direct PP has been optimized and validated.

### 3.5. Application of the Method to Real Patient Samples

**Figure 5:**
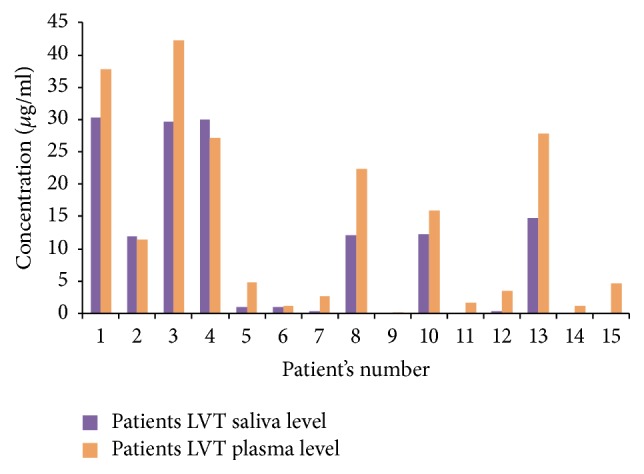
Levels of LVT in saliva determined by the proposed HPLC method and in plasma determined by a standard SPE-HPLC-DAD.

The method was applied for determination of LVT in real patients (children) who have been prescribed LVT, and results were compared to those obtained in plasma as summarized in Figure 5. Accordingly, Levels of LVT in saliva were generally lower than the corresponding values in plasma (average ratio = 61%) and thus being intermediate between the two reported values by previous studies (36–41%, [21]) and 100% [22]. Considering the average reported therapeutic range of LVT as 10–50 *μ*g/ml [4], and the corresponding saliva range to be 40% (as a minimum) of that range then the minimum therapeutic concentration in saliva could be estimated as 4 *μ*g/ml. According to the data in Figure 5 at least 8 out of 15 patients had concentrations in saliva less than the estimated lowest therapeutic level of 4 *μ*g/ml, and 4 of these patients (>25%) had levels of LVT bellow LOQ of 0.24 *μ*g/ml. In these patients, plasma LVT levels were also below the reported minimum therapeutic concentration (10 *μ*g/ml) which is an alarming finding that such a high percentage of patients were not receiving the minimum therapeutic concentration. Although some of the observed under optimal therapeutic levels of LVT might be attributed to interindividual variation in metabolic rates, absorption and elimination of the drug and dosing level, patients compliance seems to be a key point. Surely the extent of patient's compliance is expected to have seriously contributed to the observed low concentrations of LVT. The general patients compliance rates for epileptic patients on different antiepileptic drugs have been estimated to be <75% [26].

**Figure 6:**
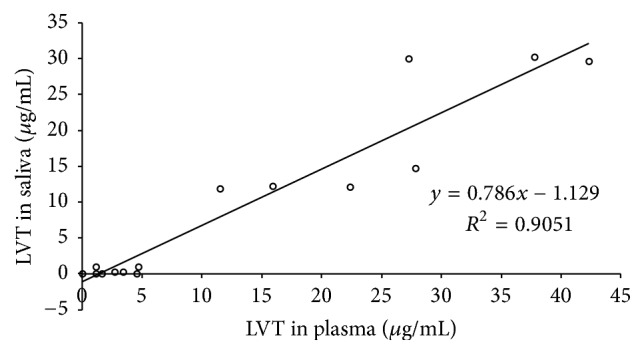
Correlation between LVT levels in saliva determined by the proposed PP-HPLC-UV method and that in plasma determined by SPE-HPLC-DAD.

To further evaluate potential correlation between plasma and saliva levels of LVT, the obtained concentration values in both matrices were plot in Figure 6. A reasonable correlation with a correlation coefficient of 0.9 was obtained, being close to the only two values (0.87 and 0.9) that were previously reported [21, 22]. Therefore, saliva was shown again as a practically usable sample to monitor the levels of LVT in patients for the purpose of improving therapeutic response or patient's compliance. Indeed, the data presented here reveals significant proportion of children who were prescribed the drug but exhibited levels (in saliva and plasma) below the estimated therapeutic ranges. Performing therapeutic drug monitoring through saliva is expected to be more effective so that the parents of patients may take the samples themselves and send them to laboratory for analysis.

**Figure 7:**
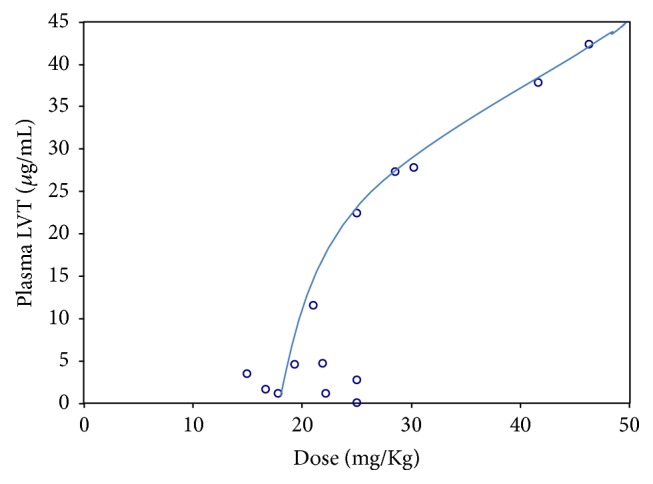
Plot of the obtained plasma levels for LVT against administered dose (the line is a manual fitting of the data).

**Figure 8:**
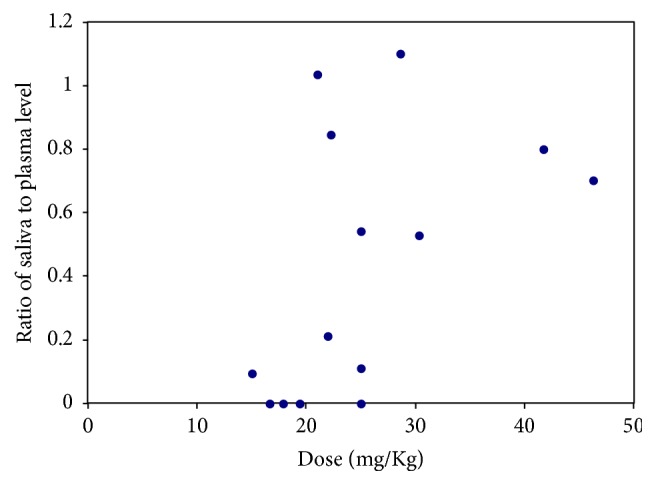
A plot of the obtained ratio of LVT in saliva to plasma against the administered dose.

In order to investigate if the degree of correlation was dependent on the administered dose and level of LVT in plasma, the obtained levels of LVT in plasma were plot against the prescribed dose of LVT as mg/kg (Figure 7). Although the obtained concentrations in plasma were obviously proportional with the prescribed dose, the relationship was curvilinear rather than a linear relationship that was reported in previous studies [22]. The fact that the involved patient's group was of children only (2–14 yr) might have contributed to the observed concentration/dose profile. It is obvious that the curve tends to have a different slope at doses > 30 mg/kg; reflecting a change in the net rate of absorption/metabolism/elimination process. The observed change in slope of the curve at doses > 30 mg/kg might influence the nature of correlation between plasma and salivary levels. Interestingly, the correlation between salivary and plasma levels appeared to be improved at higher administered dose s as shown in Figure 8, where the ratios between saliva and plasma levels were closer to 1 in high administered doses but not in the low doses patients. A plausible explanation of the observed pattern of correlation accords with the presence of a threshold mechanism where LVT can be secreted in saliva after reaching certain level in plasma. Accordingly, the good correlation is mostly anticipated for patients at higher doses of the drug, that is, >30 mg/kg which represent a limitation of the approach.

## 4. Conclusion

After assessing different columns with different packing materials as stationary phases, C8 stationary packing material was shown to be clearly superior to other more hydrophilic stationary phases with regard to having adequate retention of LVT. Other types of stationary phases did not provide sufficient retention for LVT and thus were shown to be of lower ability to separate LVT from saliva constituents. Amine modifiers in the mobile phase were shown to have no effect on retention or peak shape of LVT. Sample solvent, however, appeared to have a major effect on peak shape and response; namely, the solvent must not be significantly stronger than the mobile phase. Having optimized both stationary phase and mobile phase, the developed method in this study, which is the first isocratic method to be reported for determination of LVT in saliva, provided excellent selectivity, precision, accuracy, and a satisfactory LOQ.

Simple PP was found adequate to provide high recovery of LVT from saliva with a reasonably clean sample that allowed satisfactory separation of LVT from other natural saliva constituents. Optimization of all relevant parameters in light of literature reports and the anticipated behavior of LVT based on its physicochemical properties led to excellent performance in terms of selectivity, recovery, and sensitivity. For example, maximizing retention of the analyte has led to quite high selectivity and the use of simple PP for sample preparation allowed easy procedure together with maximum recovery based on the knowledge that LVT is a highly hydrophilic and non-protein binding drug.

Application of the optimized method to saliva samples taken from real patients on LVT revealed a reasonable correlation between the measured LVT concentrations in plasma and saliva. Although the correlation of LVT concentration in plasma and saliva has been studied and shown in previous studies, our study is the first to confirm the existence of correlation in a group of children patients. Over a third of the studied patients sample demonstrated alarming plasma and salivary LVT concentrations which were below therapeutic range. The simple and accurate method developed for determination of LVT in saliva should facilitate frequent checking of salivary LVT levels which were shown to be reasonably correlated with plasma levels.

## Figures and Tables

**Table 1:** Summary of recovery, accuracy and precision data obtained for the proposed method (*n* = 5).

QC sample (*µ*g/ml)	Recovery	Accuracy (% error)	Precision (RSD)
Low (0.24)	98.2%	4.2%	7.6%
Medium (18)	98.5%	2.7%	5.2%
High (48)	99.6%	1.6%	4.3%

**Table 2:** Comparison of analytical performance of the proposed method with relevant published methods.

Validation parameter	Proposed method	Guo et al., 2007 LC-MS/MS	Mecarelli et al., 2007 GC-MS
LOQ (*µ*g/ml)	0.24	1^*∗*^	1.1^*∗*^
Linearity (*r*)	>0.992	NA	0.998
Precision (RSD)	7.6–4.3%	8.2–5.3%	13–2%
Recovery	98.2–99.6	103–108	NA
Accuracy (% error)	4.2–1.6%	Judged by recovery	17.9–0.3%

^*∗*^Lowest concentration on calibration curve.
